# Partial Shocks on Cooperative Multiplex Networks with Varying Degrees of Noise

**DOI:** 10.1038/s41598-018-31960-y

**Published:** 2018-09-11

**Authors:** Keith Burghardt, Zeev Maoz

**Affiliations:** 1 Information Sciences Institute, University of Southern California, Marina del Rey, California, 90292 USA; 2 Department of Political Science, University of California, Davis, Davis, California, 95616 USA

## Abstract

In many cooperative networks, such as alliance and trade networks, abrupt and intense changes to the state of the system (which we call “shocks”), can substantially change the network. We examine how such shocks affect multiplex networks via an agent-based model, in which agents add, drop, or change ties to increase their utility. At a certain time-point, some agents are “shocked” by changing (increasing or decreasing) the cost associated with tie-formation or tie-maintenance. Our model makes several improvements to previous models, including (a) only a fraction of nodes are shocked to simulate small wars or scattered tariff increases or decreases and (b) agents can make both utility-maximizing decisions and randomly rewire ties to explore the utility landscape. Interestingly, we find that randomly rewiring ties increases the utility of agents, for reasons similar to simulated annealing in physics. Furthermore, we create a novel metric to determine how networks change after a shock and find that the size of a shock and noise significantly changes the network, but only when agents’ incentives for tie-formation are sufficiently high. Together, these results suggest that adding more realism to cooperation network models can give nuanced understanding to network shocks.

## Introduction

Social, political, economic and organizational networks often emerge from individual incentives to form ties within and across functional domains, i.e., to cooperate^1–4^. Examples include international trade ties, security alliances, friendship networks and organizational ties. These networks emerge because ties serve certain functions; ties form because they benefit the agents associated with them. While each agent in a network may have different reasons for forming ties with other nodes, the collection of ties yield common emergent structural patterns^5,6^. For example, networks exhibit transitivity, in which allies of allies are allies^6^. Likewise, international trade networks form trading clusters out of tendencies of states to share trading partners with other states^7–9^. These patterns are similar to other social processes, such as homophily and following social norms^10–12^. We also find that many cooperative networks are coupled such that changes in one induce changes in the other, which we call “spillover”. This implies that seemingly isolated shocks can propagate from one network layer to other network layers within a multiplex network forcing multiple network layers to adapt^4,13–18^. For example, wars may affect the structure of alliance networks and propagate from alliances to trade^7,19–21^. Finally, real-world networks have a large distribution of ties, such that many agents have a few ties, but also a few agents have ties to many other agents. Non-trivial tie distributions are also a strong indicator of non-trivial mechanisms that underlie the formation of a network^5^.

If network ties serve certain functions, then we can reasonably assume that agents use these ties to maximize their individual utility. Under this assumption^22^, we can create a minimal model to explain the structural attributes of such networks. This model is not meant to reproduce the “true” utility function, or incentives, of a particular social system, but instead provides a simple framework and therefore complimentary to previous models in other fields^23–25^.

**Figure 1:**
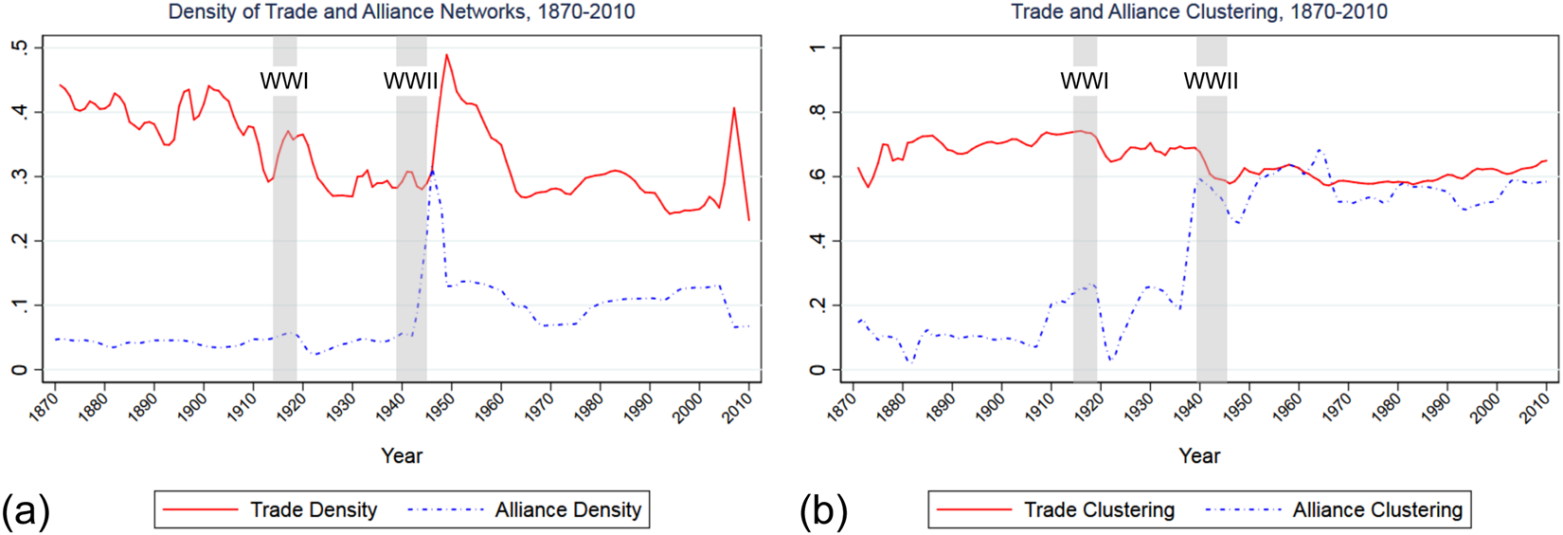
Network density and clustering of alliance and trade networks, 1870–2010. Shaded areas represent World War I and World War II, respectively.

Cooperative networks may undergo shocks: they experience radical changes in the factors that affect nodal decisions about forming, retaining or dropping ties, as well as evolve in longer timescales^1,3,4,15,26^. This is similar to how networks adapt to the spread of diseases^17^. In some cases, networks retain their pre-shock characteristics; in other cases, shocks cause phase transitions that fundamentally alter the structure of these networks. To illustrate how shocks induce changes in real-world networks, we show in Fig. 1 some of the key characteristics of two international networks: an alliance network^7,27,28^ and a trade network^29^. An unweighted tie is defined as any alliance, or any significant amount of trade between countries, respectively. Significant trade between nodes *i* and *j* is defined as trade (imports plus exports) greater than 0.5% of country *i*’s total trade.

Figure 1 shows how some political events can have lasting effects on the structure of social and political networks. Large-scale wars—especially World War II—result in dramatic spikes in the density of alliances, followed by a return to a more stable level but at a generally higher density than in the previous period. The trade network density spikes after World War II, possibly as a consequence of the Bretton Woods Treaty and the institutionalization of monetary and trade practices. But it returns to “normal” levels in the 1970s, around the time the U.S. eliminates the gold standard. Another noticeable spike took place in the first seven years of the 21st Century, followed by a sharp drop, probably as a result of the global recession. The two World Wars furthermore had a dramatic effect on alliance clustering, especially after World War II. By contrast, trade networks maintained a relatively high level of clustering without any major changes. Accordingly, under the assumption that agents, such as countries, form ties to maximize their utility, we are interested in studying several questions:

1. How do networks evolve given different tie-formation and tie-maintenance incentives?
2. How do networks react to shocks that cause drastic changes in the factors that affect agents’ utility?
3. How do shocks affect unshocked agents? To what extent do the effects of shocks on shocked agents differ from the effects of shocks on unshocked agents?
4. How does noise, defined as non-utility-maximizing decisions, affect network structure?

To answer these questions, we extend upon an agent-based model (ABM) that simulates (a) multiplex network formation under a set of conditions that may mimic the emergence of cooperative networks out of a set of individual utility-maximizing decisions to form ties with each other, (b) the occurrence of shocks in a subset of agents, defined as drastic changes in their utility function, (c) processes of multiplex network reorganization following these shocks, and (d) how networks evolve when agents choose to explore their utility function by sometimes rewiring their ties at random.

## Methods

We adopt the utility function spelled out by^30^ (following^31^). As in the previous study^30^, we assume a multiplex network composed of two layers. This helps us understand how networks interact with each other^19,32,33^. The current study expands on the previous one by focusing on *partial* shocks, which are shocks that affect only a fraction of the agents. This contrasts with *systemic* shocks, which are shocks that affect all agents, the focus of ^30^. Furthermore, we explore in greater depth how rewiring ties affects network evolution. This allows us to examine aspects of network reorganization that were not possible given systemic shocks. We elaborate on this point below.

The structure of the ABM is straightforward. There are two layers, for example, professional ties and friendship ties, or alliance ties and trade ties. Agents can form ties in any given layer. Let each agent, *i*, have an associated utility, *u*_*i*_, for the ties it currently has. Then:

1 $${u}_{i}=e{v}_{i}+\sum _{\ell \in \mathrm{\{1},\mathrm{2\}}}\,(b{t}_{i\ell }-{c}_{i}{t}_{i\ell }^{2}+d{z}_{i\ell }),$$

where $\ell$ indexes the layer of the multiplex network, ${t}_{i,\ell }$ is the number of ties of agent *i* in layer $\ell$, *z* is the number of closed triangles associated with the agent (neighbors of neighbors are neighbors) and *v* is the number of spillover ties between agents *i* and all agents *j*. Finally, *c*_*i*_, *d* and *e* are parameter values that weigh each factor in the utility function and therefore indicate by how much one’s utility increases (or decreases) as a function of the number of ties, closed triangles and spillover ties, respectively.

The motivation for this utility function is as follows. We assume a uniform payoff, $b{t}_{i\ell }$ for each tie, in addition to a cost, ${c}_{i}{t}_{i\ell }^{2}$, to better represent real networks, such as alliance and trade networks^34^, where nodes do not all connect to each other. Similarly, there is a payoff for spillover ties to form, which we define as *ev*_*i*_, to explain why they appear more often than chance^19^. Finally, to explain the significant clustering in real systems, seen in Fig. 1 and^6^, we provide a payoff for triangles in each layer, $d{z}_{i\ell }$. The utilities are additive, versus multiplicative, for simplicity. If all payoffs were multiplicative, for example, then the log of utility would trivially have the same form and the utility-maximizing decision in the current equation or in the multiplicative form would be the same.

After a time *t*_*s*_, some agents are shocked, meaning their tie cost either increases or decreases:

2 $${u}_{i}=\{\begin{array}{cc}{u}_{i,us} & t < {t}_{s}\\ {u}_{i,s} & t\ge {t}_{s},\end{array}$$

where

3 $${u}_{i,s}=e{v}_{i}+\sum _{\ell \in \mathrm{\{1},\mathrm{2\}}}\,(b{t}_{i\ell }-{c}_{i,s}{t}_{i\ell }^{2}+d{z}_{i\ell }).$$

In our simulations, *c*_*i*_ = 0.2 (“low”) or 0.6 (“high”) and *c*_*i*,*s*_ = 0.2 if the initial cost, *c*_*i*,0_ = 0.6 (“low-to-high” or LH shocks), otherwise *c*_*i*,*s*_ = 0.6 if the initial cost, *c*_*i*,0_ = 0.2 (“high-to-low” or HL shocks), which is similar to previous work^30^. We chose these specific values of *c*_*i*_ because they reflect threshold values for low and high cost. When *c*_*i*_ = 0.2 agents have an incentive to form a large number of ties (for example with *d* = 0.3, *e* = 0.3 and *c*_*i*_ = 0.2, the optimal degree of a node is 9 in one layer and zero in another, yielding a utility score of 3.6; for the same values of *d* and *e*, but with *c*_*i*_ = 0.6, the optimal degree is 1 in one layer and zero in the other, yielding a utility of 0.4). Other values for *c*_*i*_ should not affect our qualitative findings. Unlike previous research^30^, we study what happens when only a subset of nodes are shocked, which allows us to examine how shocked agents and unshocked agents interact with each other and how shocks spread outwards to previously unaffected agents. This reflects, for example, how some people who become parents may have less time to spend with friends. States that increase tariffs may raise the cost of trade for actual or would-be trading partners. A dramatic rise in oil prices may increase the costs of transportation, which may make certain contacts less attractive. By contrast, major technological innovations may reduce significantly the cost of forming ties. For example, social networking programs such as Twitter or Facebook may enable people to communicate online and therefore they need to spend less time in physical contact with friends, which may lead to more friendship ties.

In our paper, agents add ties, remove ties, or rewire (both add and remove a tie) in order to maximize their utility. This is in contrast to a previous paper in which agents did each step in order without determining which move was utility-maximizing^30^. Furthermore, we allow for agents to make several tie offers, or tie drops, at once, to simulate multilateral trade agreements, alliances, or social networks, where an agent may choose to make multiple ties at a given time. In some cases, networks can equilibrate when no agent finds it beneficial to change ties, or no new tie-offers are accepted. Importantly, we assume that tie-formation requires bilateral consent: both agents have to agree (for example, because both improve their utility) for a tie to form. However, ties can be dissolved unilaterally. If an agent believes that one or more current ties adversely affect its utility, it can drop them without the consent of its neighbors.

When tie costs increase, agents will see their utility drop, as the increased tie-costs make the existing ties excessively expensive and additional tie-formation becomes counterproductive. In such situations, rational agents must drop some of their ties to maximize their utility. If tie-costs suddenly drop, some agents are faced with new opportunities for tie-formation that did not exist before the shock. In that case, they may find it beneficial to add ties, as they might improve each agent’s utility.

We model agents’ behavior via the following algorithm:

1. Agent *i* is picked at random from a pool of *N* agents. In the main text, *N* = 40, but larger N, such as *N* = 100, produces quantitatively similar results (see Supplementary Figs S1, S2, S3 and S4).
2. With probability 1 − *p*, repeat *N* times:
  - *i* finds the utility of making ties with *m* ≤ *N* − 1 individuals at random.
  - Next, the utility of dropping each tie is found.
  - Finally, the utility of simultaneously adding and dropping a tie (“rewiring”) is found, following the same algorithm as adding a tie.
  - Agent *i* maximizes their utility by either doing nothing, offering a tie, dropping a tie, or rewiring.
  - With probability 1 − *p*, an agent who is offered a tie will accept it only if it increases their utility. Otherwise, with probability *p* an agent who is offered a tie will accept a tie regardless of its effect on a node’s utility at this timestep.
  - Time *t* → t + 1/*N*.
3. With probability *p*, do once: a random tie is created and another is dropped at random and time *t* → *t* + 1/*N*.

We choose *N* to represent the approximate number of agents in alliance and trade networks. We also assume that the utility of *m* agents are chosen at random, to make the fewest assumptions about how agents are chosen. In the Supplementary Text, we also create a “smart” method to choose agents for utility maximization in which agents attempt to assess the utility of their neighbors (to establish spillover ties) and the utility associated with the neighbors of their neighbors (to explore the value of closing triangles) rather than to assess the utility of ties to randomly selected agents but the results are qualitatively similar (see Supplementary Figures S1, S2, S3 and S4). After the network reaches approximate equilibrium (*t*_*s*_ = 50 timesteps), a shock is applied to *f* ≤ *N* agents. For each “shocked” agent, tie-cost, *c*_*i*_, either increase or decrease, as mentioned above.

We assume that agents behave in a way that tends to create short-term gain, although more realistic models in the future may allow for agents to make decisions in which they anticipate the behavior of other agents. We observe the network at timestep 100 after creating a shock just after timestep 50. For the rest of the paper, agents check the utility of offering ties to *m* = 10 other nodes, but the number of nodes shocked, *f*, vary from *N*/3 to *N*, noise, *p*, varies from 0 to 0.75, while the triangle payoff, *d* and spillover payoff, *e*, vary from 0 to 2. We ignore *p* = 1.0, because the network will trivially evolve independent of the utility function and, because links rewiring at random would create an Erdös-Renyi-like network. In summary, we have 3 parameters in the utility function, the cost, *c*_*i*_, triangle payoff, *d* and spillover payoff, *e*, that we vary and two in the agent algorithm: the fraction of nodes that are shocked, *f* and the noise, *p*.

Smaldino *et al*. found that networks have emergent “memory” (or what they call “structural entrenchment”)^30^ when shocks are systemic and there is no noise (*p* = 0). In other words, when the payoffs associated with certain types of ties (e.g., closed triangles, spillover ties) are sufficiently high, “shocked” networks become very resilient; that is, they retain a structure that is not much different from “non-shocked” networks (or from the pre-shock networks).

We extend the previous study by focusing on several additional issues. First, we assess network resilience using a novel metric that determines the degree to which utility and several network topological metrics are preserved. Second, we assess the effect of indirect shocks. Specifically we study how shocks in the system affect non-shocked agents and how these effects differ from the manner in which shocked nodes react to changes in tie-costs. Third, we explore in greater detail how randomly rewiring links can affect network structure, which is modeled by *p* > 0.

## Results

### Network Resilience

We start by comparing the key network characteristics under different levels of shock and noise. In previous work, a resilience statistic was defined for LH system shocks^30^. In the current study, however, we use a novel metric for shocks to determine (a) the difference between shocks that involve an increase in tie-costs (and thus an expected decrease in connectivity) and shocks that involve a drop in tie-cost (and are expected to result in increased connectivity), (b) the effect of varying the number of nodes shocked and (c) the effect of shocks on several network statistics. The metrics can be written as:

4 $$\delta =1-\frac{N{S}_{0}-N{S}_{s}}{N{S}_{LL}}$$

5 $$\theta =\frac{N{S}_{s}-N{S}_{0}}{N{S}_{LL}}$$

where *NS*_0_ is the pre-shock value of given network statistic (defined below), *NS*_*s*_ is the post-shock value of the same statistic and *NS*_*LL*_ is the average value of the low-cost (LL) control condition at timestep 50. The resilience metric *δ* reflects the degree to which a given node (or the network as a whole) retains its properties following a LH shock, compared to its pre-shock properties and to the relevant control condition. By contrast the flexibility *θ* reflects the degree to which a network incentivized by a HL shock to change, approximates the network property that would have obtained had the network started forming with a low cost.

In this paper, we specifically focus on mean degree, mean clustering coefficient^6^ and mean utility at the last timestep. In addition, because our study reflects co-evolution of a two-layered multiplex network and because the utility function contains a spillover payoff, we create a new metric called mean fraction spillover. Fraction spillover for a given node is defined as:

6 $${S}_{i}=2\frac{\sum _{j}\,\prod _{\ell \in \mathrm{1,2}}{a}_{ij\ell }}{\sum _{j}\sum _{\ell \in \mathrm{1,2}}\,{a}_{ij\ell }}$$

where $\ell$ indexes the layers of the multiplex network and the adjacency matrix, ${a}_{ij\ell }$, is assigned a value of 1 when an edge exists between nodes *i* and *j* in layer $\ell$ and zero otherwise. This statistic measures the fraction of a node’s total degree that overlap across both layers of the multiplex network. Because the utility function encourages spillover, this metric allows us to determine the degree to which spillover increase in the network versus the spillover payoff, *e*.

**Figure 2:**
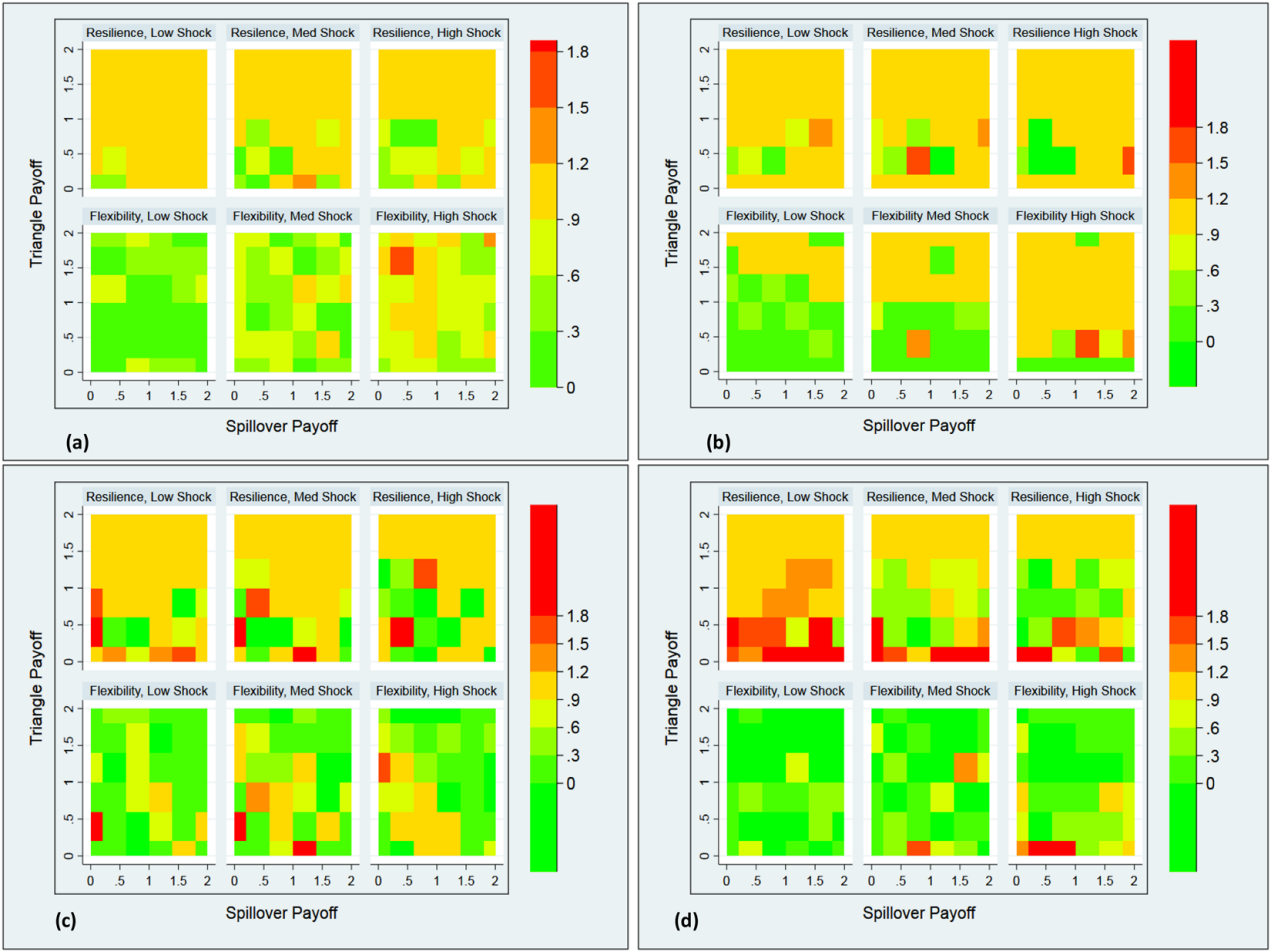
Network resilience and flexibility versus the number of nodes shocked in simulations with no noise (*p* = 0). (**a**) degree resilience/flexibility, (**b**) clustering resilience/flexibility, (**c**) spillover resilience/flexibility, (**d**) utility resilience/flexibility. Low shock: 13 nodes shocked, medium shock: 26 nodes shocked and high shock: all (40) nodes shocked.

Figure 2 displays resilience (upper panel of each subfigure) and flexibility (lower panel of each subfigure) of various network statistics as a function of spillover payoffs, triangle payoffs and number of nodes shocked, with noise set to zero. Three important results emerge. First, as expected, resilience levels decline and flexibility scores increase with more shocked nodes. Second, degree, clustering and utility resilience appear to be more sensitive to triangle payoffs than to spillover payoffs. The same holds for degree and clustering flexibility. Interestingly, the resilience and flexibility of spillover ties is roughly as sensitive to triangle payoffs as it is to spillover payoffs. This suggests that even when agents are rewarded for forming spillover ties, they prefer forming them if such ties also tend to close triangles.

Overall, our results generalize the results of the previous study^30^ to varying the number of nodes shocked using a plausibly more realistic simulation of the dynamics. We find that sufficiently high incentives to form ties increase both resilience and flexibility and that this effect is sustained for partial as well as for systemic shocks.

### Noise

We now examine the effects of noise on network structure. Recall that the noise parameter, *p*, represents the fraction of decisions that are made randomly, as opposed to utility-maximizing. We can interpret this as agents exploring the utility landscape and changing the network in order to find network structures that increase utility most. We varied *p* from 0.0 (all decisions are utility-maximizing) to 0.75 (three-quarters of all moves are rewiring links at random).

**Figure 3:**
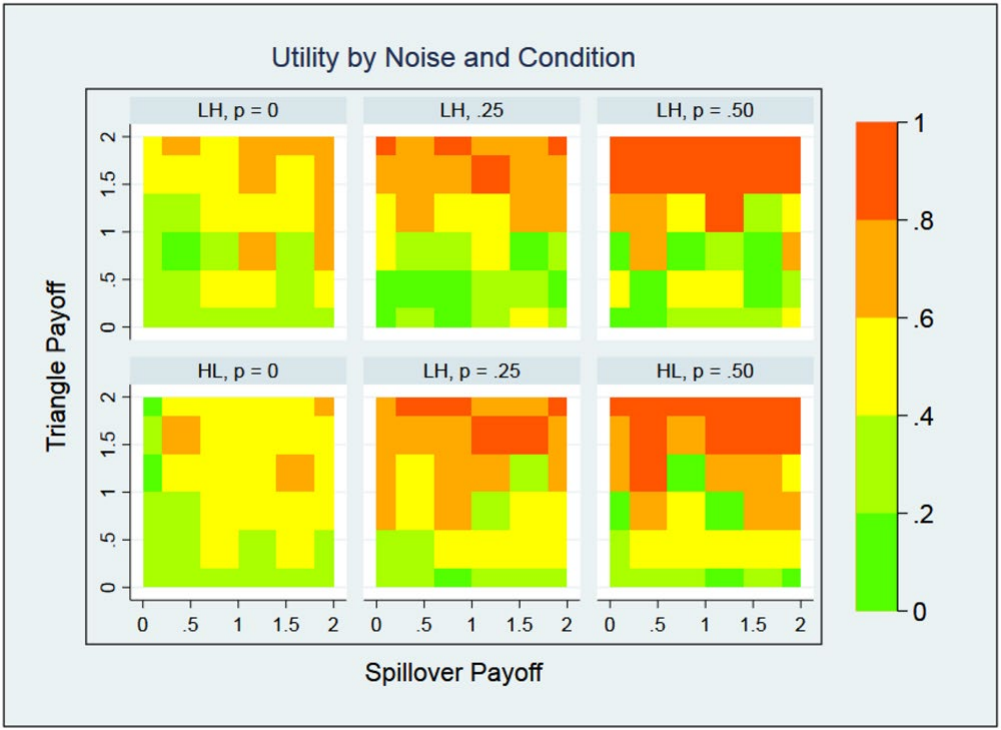
Network utility by noise and conditions. Noise levels are limited to 0 ≤ *p* ≤ 0.5 and 26 nodes are shocked. Results are similar for other values of noise and numbers of shocked nodes.

Figure 3 displays the effect of noise on utility and demonstrates that, regardless of the type of shock, utility often increases with noise. Moreover, the increase in utility seems to be insensitive to spillover and it has a threshold value of *d* ≈ 1 for triangle payoff, which is similar to the threshold predicted in^30^. This results seems to defy intuition. Agents are simulated to make optimal, utility-maximizing decisions. Tie-formation, tie-maintenance, tie-dropping, or rewiring should, at first sight, yield the highest level of utility when noise level is zero, when no agent attempts to reduce their utility. What we observe is just the opposite of this expectation: the higher the fraction of random tie-making, rewiring, or tie-dropping decisions, the higher the utility of individual agents as well as the overall network utility (what Jackson and Wolinsky^22^, call “efficiency”). However, there is a perfectly reasonable, if somewhat counterintuitive, explanation of this result. In our model, agents are simulated to make myopic decisions. Specifically, each agent selects the “best” (utility-maximizing) tie-formation, tie-dropping, or rewiring alternative in terms of the immediate utility it gets following its own move. An agent will avoid a move that causes it to lose utility at that particular time-point, even though this move may cause it to increase its utility later due to choices of other agents.

**Figure 4:**
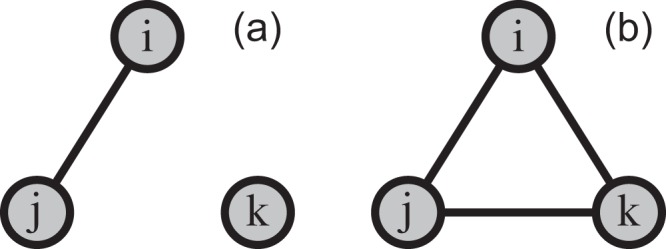
Clustering for one layer of our model. (**a**) The network that forms when the tie cost is high, starting from a isolated nodes without noise (*p* = 0). (**b**) If *d* > d^*^ = 0.8, this network is the global utility maxima.

Consider a typical scenario represented by Fig. 4. In this figure we assume a high tie-cost. In Fig. 4a, agents *i* and *j* are connected. For each of them, when there is no noise and they optimize their utility at the next time-point, adding a tie with *k* (or accepting a tie-offer from *k*), yields negative utility. Hence the network in Fig. 4a equilibrates at that particular time-point. Agents *i* and *j* do not have an incentive to form a tie with *k* when it is their turn to move. Agent *k* has an incentive to form a tie with either of the other agents, but its tie-offers will be rejected by the latter agents. However, when there is some level of noise, *k* would (rationally) offer a tie to either *i* or *j* and either agent might accept (which lowers their utility in the short term). In the next step, if triangle payoff is sufficiently high, the third node has now a rational incentive to close the triangle, as shown in Fig. 4b. Consequently, the network equilibrates in that state. All nodes increase their utilities at the Pareto-optimal equilibrium. Hence, networks tend to equilibrate at Pareto-inferior states under low noise, while the same networks tend to become denser as noise increases and the equilibrium reflects a higher clustering, as shown by the threshold effect of triangle payoffs on utility. Surprisingly, this is similar in spirit to simulated annealing in physics^35^.

In traditional simulated annealing, simulated atoms can either change their state to minimize energy, or with a particular probability, they can change their state at random, which may locally increase energy (when the temperature, the parameter that controls the amount of “noise” in the system, is non-zero). What is non-trivial is that a non-zero temperature allows the system to explore the energy landscape so that it does not get caught in what is known as local minima. Eventually, it can reach either a deep local minimum or a global energy minimum and the state is in a much lower energy than if the temperature was zero (in which atoms changed state such that their energy strictly decreases or stays the same). Similarly, agents in our model are allowed to either increase their utility or rewire at random (therefore making choices that are locally not utility-maximizing). Agents eventually self-organize to create network structures with far higher mean utilities than the noiseless (strictly utility-maximizing) algorithm.

**Figure 5:**
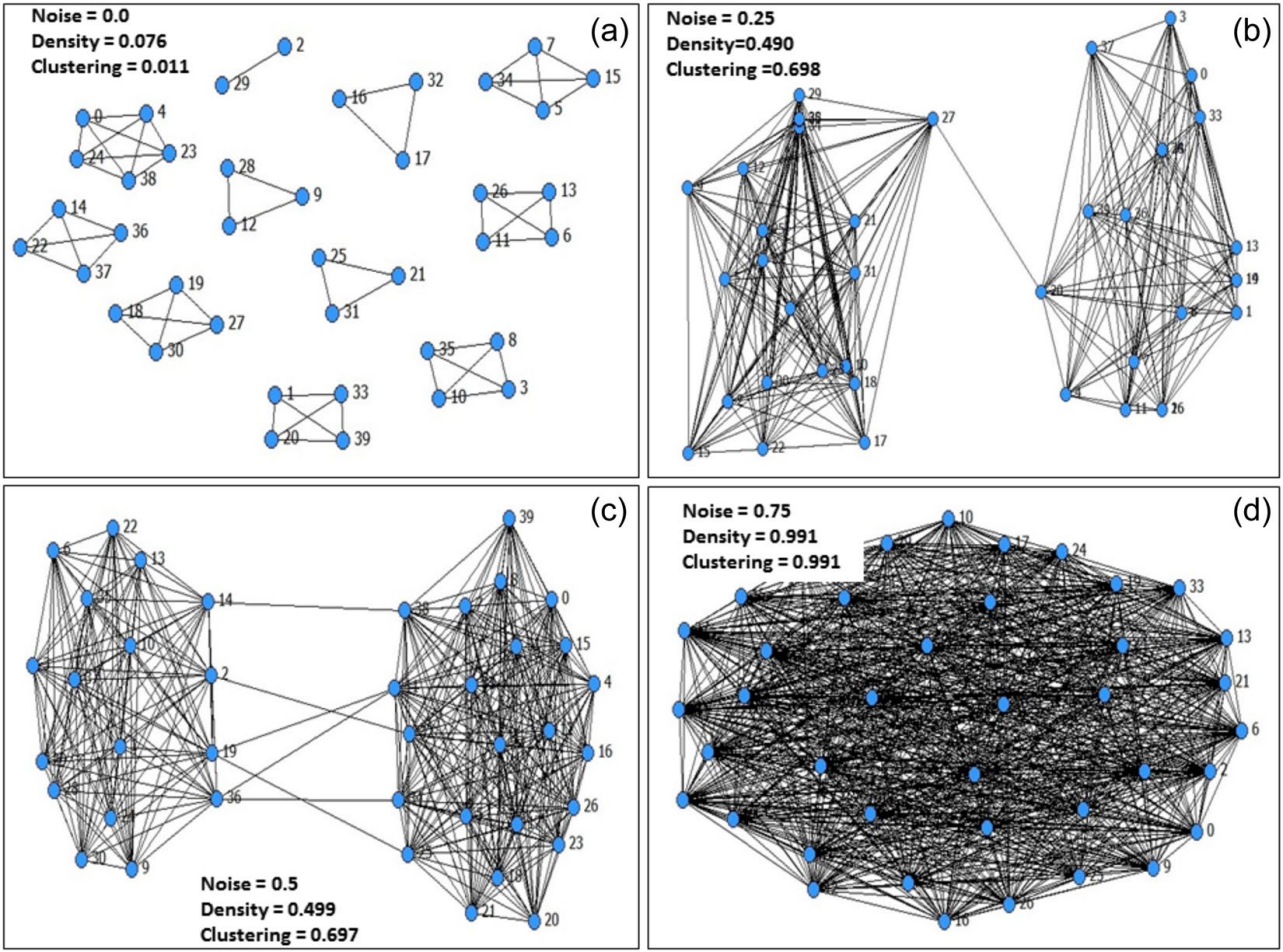
Example networks for varying amounts of noise (*d* = *e* = 1.2) where *N* = 40. Next to each network is the average density and clustering coefficient.

Figure 5 shows a typical scenario of the way in which noise affects connectivity and clustering. In this figure, a typical network that forms with a set of fixed parameter values becomes increasingly connected and clustered as noise increases. The implication is that, in cooperative interactions, myopically rational agents can sometimes benefit from links randomly rewiring to simulate agents exploring their utility landscape. This result has potential implications for a wide array of cooperative networks in the real world. Interestingly, we also find that agents take longer to equilibrate with the addition of noise. For example, while no noise produces fast convergence to equilibrium, high noise when tie costs are high and low noise when the tie costs are low, produces the largest increases in utility between timestep 50 and timestep 100 (see Supplementary Table S1). The reason for this counter-intuitive finding requires further study.

### Determinants of Network Statistics

**Figure 6:**
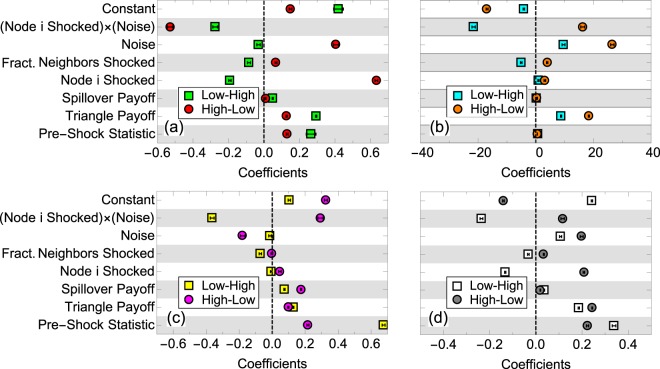
Marginal effects of OLS model for (**a**) clustering, (**b**) degree, (**c**) fraction of spillover for LH and HL shocks and (**d**) utility.

In this section, we discuss the marginal determinants of network structure at the nodal level. We ran a number of mixed effect ordinary least squared (OLS) regressions on node degree, local clustering coefficient, fraction spillover and utility, as seen in Fig. 6. To reduce the effect of outliers, we normalize utility by its cumulative distribution function: *u*_*norm*_ = *P*(*u* ≤ *u*_*i*_), where *P*(*u*) is the probability of a given value of *u* across all conditions and all timesteps. A normalized utility of 0.5 is therefore the median utility value across all conditions. For each node, *i* and each node statistic, *NS*_*s*,*i*_, the model is defined as:

7 $$N{S}_{s,i}=a+{b}_{1}N{S}_{0,i}+{b}_{2}({s}_{i}\times p)+{b}_{3}p+{b}_{4}({f}_{n,i})+{b}_{5}{s}_{i}+{b}_{6}e+{b}_{7}d+{\varepsilon }_{i}$$

where *a* is a constant, *b*_*j*_ are coefficients, *NS*_0,*i*_ is the pre-shock node statistic, *s*_*i*_ is the shock indicator function, *p* is noise, *e* is the spillover payoff, *d* is the triangle payoff and *ε*_*i*_ is the error term (not to be confused with noise, which is a model parameter). Finally, *f*_*n*,*i*_ is the fraction of neighbors shocked:

8 $${f}_{n,i}=\frac{\sum _{\ell ,j\ne i}\,{a}_{ij\ell }{s}_{j}}{\sum _{\ell ,j\ne i}\,{a}_{ij\ell }},$$

where ${a}_{ij\ell }$ is the adjacency matrix for each layer just before a shock and *s*_*j*_ is the shock indicator function. Notably, degree heterogeneity, seen, for example in Fig. 5, is a natural property of our model and is seen in real networks as well^5^. Quantitative similarities and differences between the model’s degree heterogeneity and real data will be explored in future work.

First, we find that the pre-shock values of a given network statistic have a strong impact on the post-shock values of that statistic, regardless of the shock type. Nodes that had high degree, high clustering, or high utility in the pre-shock equilibrium tend to maintain high levels of these attributes. This is the case whether the shock is of the LH type, thereby forcing nodes to drop some ties, or whether it is of the HL types, incentivizing them to add ties.

Second, both triangle and spillover payoffs have a consistent effect on network attributes, regardless of the shock type. However, while the effect of triangle payoffs on post-shock network statistics is robust across shock types and across network statistics, the effects of spillover payoffs is less robust and, judging by the beta coefficients, generally weaker than the effect of triangle payoffs.

The reason for that is because a single tie may close multiple triangles. A node with degree ${t}_{i\ell }$ in layer $\ell$ may have up to ${z}_{i}(max)={t}_{i\ell }({t}_{i\ell }-\mathrm{1)}/2$ triangles in a given layer. Across both layers, this may result in a payoff of $d\,{\sum }_{\ell }\,({t}_{i\ell }({t}_{i\ell }-\mathrm{1)/2})$. However, the same degrees across layers, even if all of them are spillover ties, can result only in a payoff of $e({\sum }_{\ell }{t}_{i\ell }/2)$. This was also reflected in the stronger effect of triangle payoffs on resilience in Fig. 6 above.

Whether a node has been shocked has a significant effect on its post-shock network statistics. As expected, shocked nodes in the LH condition tend to lose degree, clustering, spillover and utility. Similarly, shocked nodes in the HL condition tend to gain degree, clustering, spillover and utility. Our work differs from a previous study^30^ by focusing on partial shocks in order to evaluate how nodes are affected by shocks of neighboring nodes. We used the fraction of a node’s neighbors that were shocked as an indicator of such effects. We find that the higher the fraction of a node’s neighbors that were shocked, the lower the node’s degree, clustering, spillover and utility under the LH condition and, with the exception of the spillover case, the higher the value of the node’s post-shock network statistic under the HL condition.

Noise has inconsistent effects on network statistics. Controlling for other covariates, noise increases nodal degree and nodal utility, but it reduces nodal clustering and spillover under the LH condition. Under the HL condition, noise increases nodal degree, clustering and utility, but reduces spillover. This corresponds to the surprising effects of noise discussed above. Notably, however, pre-shock statistics are higher with noise, therefore controlling for the pre-shock statistic, the additional effect of noise is much smaller.

**Figure 7:**
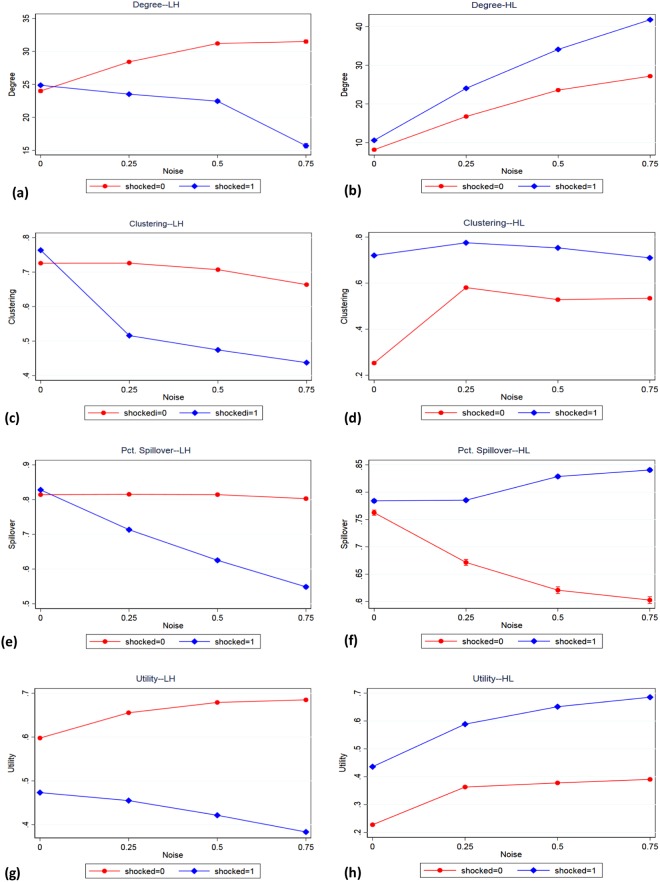
The effect of noise on degree (**a**,**b**), clustering (**c**,**d**), fraction spillover (**e**,**f**) and utility (**g**,**h**) for shocked and unshocked nodes, where 26 nodes are shocked (results are similar for other numbers of nodes shocked). LH cost shocks are shown in the left panels, while HL cost shocks are shown in the right panels. Error bars are smaller than plot markers.

In order to better understand these results, we show in Fig. 7 how the networks statistics vary with noise for shocked and unshocked nodes. We discuss first the effect of noise on nodal statistics under the LH condition. Noise has a negative impact on the degree and utility of shocked nodes but it has a positive impact on the degree and utility of unshocked nodes. Noise also has a negative effect on the clustering and on the spillover rate of shocked and unshocked nodes, but the effect of noise on shocked nodes is substantially negative, whereas the marginal effect of noise on the clustering or spillover rates of unshocked nodes is negligible or insignificant. Under the HL condition, noise increases the degree and utility of both shocked and unshocked nodes, but the slope for shocked nodes is significantly steeper (degree) or marginally steeper (utility). By contrast, shocked nodes lose some clustering, whereas unshocked gain clustering. In the case of spillover, shocked nodes increase their fraction of spillover ties, whereas unshocked nodes reduce their spillover ties.

It is instructive to understand why we observe these seemingly puzzling interaction effects. In both the LH and the HL shocks, the effect of noise on shocked nodes is straightforward. Shocked nodes are forced to drop ties in the LH condition, or they are incentivized to increase connectivity in the HL condition. So when noise is higher, their reduced (LH) or increased (HL) connectivity is due both to their rational choices and to their own random moves and their neighbors’ random moves. Consider the following scenario in a LH shock. Two unshocked nodes that did not have a tie before the shock may now have an incentive to form a tie given that some of their previous neighbors who were shocked dropped them. Their options are: (a) continue not to have a tie in the post-shock period, (b) form a tie in a single layer, or (2) form two ties, one in each layer. When *p* = 0 (the agents behave without noise), 90% of the cases where two unshocked nodes did not have a tie in the pre-shock equilibrium remain in that state at the post-shock equilibrium. However, this percentage drops to 61%, 42% and 26% with *p* = 0.25, 0.50, 0.75, respectively. Likewise, the proportion of nodes that had no closed triangle fell from 90% with *p* = 0 to 63%, 44% and 27% with *p* = 0.25, 0.50, 0.75, respectively. This explains why we see this pattern of interaction effects of shocked/unshocked nodes and noise. In the HL condition, unshocked nodes benefit from the shocked nodes’ incentive to increase connectivity. However, this tends to have strong effect on clustering but not on spillover. Unshocked nodes tend to concentrate their clustering in the layer where they had greater degree in the pre-shock period, thus reducing their spillover as a function of noise.

## Discussion

The model presented in this paper offers a stylized characterization of social network formation, network co-evolution and network reorganization following shocks. These results may offer some insights into real-world social networks that are affected by shocks. Changes in life- or family-conditions (children born, divorces, moves to different locations), may have profound effects on friendship groups. However, the pre-change structure of some groups may make them more resilient to such changes than others. In international economic settings, significant changes in import-restrictions may affect both those countries that institute such changes, their neighbors and their non-neighbors. In international political processes, states adding or dropping other states as allies may affect not only the countries that are directly involved, but also third parties that are not part of the diplomatic exchange. Moreover, since many social, political and economic networks are co-dependent, some of our results may add insight to the manner in which such dependencies are affected by various shocks. Our simulations suggest some interesting, as well as some counterintuitive results. Specifically,

1. Network resilience, the degree to which a network retains its properties following negative shocks, increases as the incentives to close triangles and to form spillover ties increase. Likewise, network flexibility, the degree to which a network matches an unshocked network, increases as a function of both clustering and spillover incentives. We also find that network resilience and flexibility are more responsive to clustering incentives than to spillover incentives. This is due to the fact that a single tie can close multiple triangles, but a single tie can only add one spillover unit.
2. Noise tends to increase network connectivity, clustering, spillover and utility for large enough values of *d* and *e*. We argue that the explanation of this counterintuitive result is the fact that “random” decisions, that is, decisions that do not maximize a node’s immediate utility, enable other nodes to create structures that benefit not only themselves, but also help those nodes who had seemingly “erred”. In other words, networks that evolve due to myopically rational decisions are benefitted by agents changing network structure to explore the utility landscape, which is analogous to simulated annealing in physics^35^. Our model is a minimal model of the real world, in which actors can be either non-myopically rational or they can explore the utility landscape, which ends up benefitting both themselves and others^36^. Brams analyzes the Cuban Missile Crisis of 1962 between the United States and the Soviet Union as a game of “chicken”. He argues that both the United States and the Soviet Union avoided a nuclear collision by acting non-myopically. Both counties were willing to absorb a short-term cost in order to prevent a nuclear confrontation, thus resolving it in a compromise that had long-lasting stabilizing effects on the Cold War.
3. Our analysis of the determinants of network structure at the nodal level shows (a) *d* increases nodal statistics, while *e* has little effect, aside from increasing the fraction spillover, (b) LH shocks reduce nodal statistics, while HL shocked increase these statistics, (c) increasing the fraction of neighbors shocked affect nodes in ways qualitatively similar to if they are shocked, *therefore shocks appear to spread to unshocked nodes*, (d) noise increases clustering and utility, but reduces degree with LH shocks and increases degree with HL shocks and reduces spillover, (e) noise levels have different effects on shocked and unshocked nodes in the LH condition, with shocked nodes losing degree, clustering, spillover and utility as a function of noise, whereas unshocked nodes tend to increase degree and utility as noise increases, (f) in HL shocks, shocked and unshocked nodes tend to increase degree.

Under a utility-maximizing assumption, our model appears to be a reasonable minimal model of cooperation in a wide variety of contexts. Agents involved in multiple forms of social interactions tend to form spillover ties and closed triangles in order to maximize their utility. Our model provides some interesting and potentially important, insights into the interaction of rational–myopic and non-myopic–processes of network formation and co-evolution. It suggests conditions that affect network resilience to shocks. And it offers ideas about how noise affects network structure both under “normal” conditions wherein networks evolve naturally and following abrupt and significant changes in tie-costs. Quantitative comparisons between this model and empirical data is left for future research.

## Electronic supplementary material

Supplementary Information

## Data Availability

All code used for simulations and basic data analyses are available on GitHub, https://github.com/KeithBurghardt/MultiplexShockCode.
