# Supplementary material for: Partial Shocks on Cooperative Multiplex Networks with Varying Degrees of Noise
Source: Sci Rep. 2018 Sep 11;8:13619. doi: 10.1038/s41598-018-31960-y (PMC6134050; doi:10.1038/s41598-018-31960-y)
Supplement: Supplementary file 1 — Supplementary Information [file 41598_2018_31960_MOESM1_ESM.pdf]

Supplementary Information: *Partial Shocks on Cooperative Multiplex Networks with Varying Degrees of Noise*

Keith Burghardt[1,*] **and Zeev Maoz**[2]

[1]Information Sciences Institute, University of Southern California, Marina del Rey, California, USA, 90292
[2]Department of Political Science, University of California, Davis, Davis, California, USA, 95616
[*]keithab@isi.edu

ABSTRACT

In this manuscript, we detail algorithm robustness checks, including increasing the system size and varying how agents maximize their utility. We first describe the alternative "smart search" algorithm, in which agents search locally for candidate ties instead of at random. This allows for agents to focus their attention on ties that may create spillover edges or close triangles, which would naively improve agent utility. We also increase the network size using the algorithm seen in the main text. In both cases behavior is qualitatively and usually quantitatively similar therefore our algorithm is not sensitive to changes in network size, and does not appear to be sensitive to alternative algorithms.

Robustness Checks

The search process in our ABM is random; agents select at random ten other agents and, for each, decide whether to add, drop a tie, or rewire (swap an existing tie with a new one). We tested a different version of this model in which agents are engaged in "smart" search. Since agents receive utility bonuses from closing triangles or forming spillover ties, they can maximize their utility by assessing the value of forming ties with the neighbors of their neighbors (closing triangles), or by forming ties with their neighbors across layers (spillover). The algorithm is as follows:

1. Agent i is picked at random from a pool of N agents.
2. With probability $1-p$, repeat N times:
   (a) For each of agent i's k_i neighbors:
      i. With probability 1/2 find the utility of making a spillover edge with that neighbor
      ii. Otherwise, find the utility of closing a triangle with a neighbor's random neighbor who is not already agent i's neighbor
   (b) If all neighbors have been exhausted, i finds the utility of making ties with $m-k_i$ agents at random.
   (c) Next, the utility of dropping each tie is found.
   (d) Finally, the utility of simultaneously adding and dropping a tie ("rewiring") is found, following the same algorithm as adding a tie.
   (e) Agent i maximizes their utility by either doing nothing, offering a tie, dropping a tie, or rewiring.
   (f) With probability $1-p$, an agent offered a tie will accept it only if it increases their utility. Otherwise, with probability p an agent offered a tie will accept a tie regardless of its effect on a node's utility at this timestep.
   (g) Time $t \to t+1/N$.
3. With probability p, do once: a random tie is created, and another is dropped at random, and time $t \to t+1/N$.

The “smart” search algorithm therefore implies that agents start by examining the utility forming connections with their neighbors such that they form spillover ties or close triangles. If they run out of neighbors, however, they choose agents at random, as before.

As noted in the text, we also test whether our results are sensitive to network size. To perform this test, we ran an identical simulation on networks of size $N = 100$. Smaldino *et al.*[1] found that resilience is not affected by network size. However, since their analyses focused on the network as a whole and were based on systemic shocks (i.e., all nodes in the network are shocked), it is possible that the changes we introduced here, i.e., variable shocks, noise levels, nodal-level analyses, would be sensitive to network size. Simulations of these sort with larger networks are computationally difficult because the simulation is found to run on $O(N^3) - O(N^4)$ time. The lower bound is because each timestep consists of N agents, and we need $O(N^2)$ time to find the triangles for each node, which are used to calculate the utility function. The upper bound is because, when p is nearly 0, we run the utility-maximizing dynamics N times. Networks that are orders of magnitude larger may be appropriate for some cooperative networks, but are substantively inappropriate for the cooperative political networks we reference, which vary between $N = 40$ and $N = 200$.

We compared the results of the random search algorithm to the results of a “smart ” search algorithm. We also compared the random-search algorithm with $N = 40$ to a random-search simulation of size $N = 100$. Figure S1 compares the utility resilience/flexibility scores of the random search algorithm (networks $N = 40$ and $N = 100$) to the parallel scores based on the “smart” search algorithm. The comparison shows that the resilience/flexibility scores are qualitatively similar.

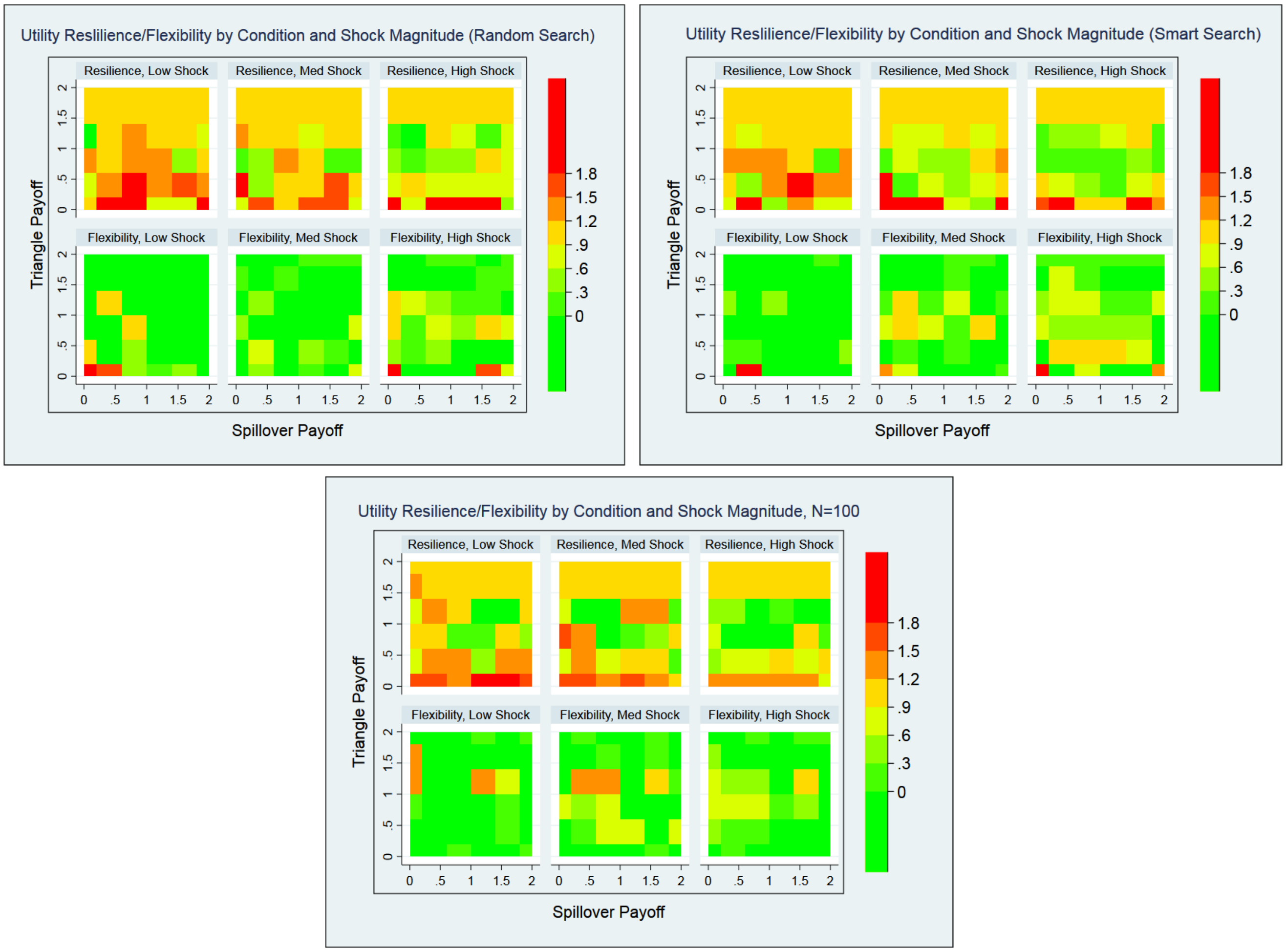

Figure S1. Random and Smart Search Comparison: Utility resilience with $p = 0$ and 26 nodes are shocked. Top layer compares simulations of size $N = 40$ with random search (left) and “smart” search (right). Bottom layer shows the results of a simulation of size $N = 100$.

We provide the results of a comparative analysis of network statistics regressed on a number of possible determinants based on three simulations: two simulations with $N = 40$, one based on random search, and one based on smart search, and a

larger simulations (N = 100) based on a random search algorithm). The results suggest that, with few exceptions, the effects of utility parameter values (d, e), shocks, neighborhood shocks, noise (p), and the interaction between shocked nodes and noise on network statistics are highly similar. It appears therefore, that our conclusions are robust to search type and network size. For completeness, we also show main text Fig. 2 for $N = 100$ (Fig. S3) and main text Fig. 7 for $N = 100$ (Fig. S4) and find results are qualitatively the same, therefore our results do not appear to be strongly dependent on network size.

Finally, as noted in the text, we compare the difference in utility scores in the final (T100) time-point of the simulation to the utility scores midway through the simulation (T50) for the LL and HH conditions. Table S1 shows the results of this analysis. When $p = 0$ all networks equilibrate in both the LL and HH. condition. However, they do not generally equilibrate when $p > 0$. Moreover, the differences between noise levels are highly significant ($F_{3,86,396} = 655.13$ for the LL condition and $F_{3,86,396} = 1,208.92$ for the HH condition). As can be seen, utility differences are maximized at $p = 0.25$ in the LL condition and at $p = 0.75$ at the HH condition. The reason for the difference between the p parameter for the LL and HH conditions is not, as of yet, entirely understood.

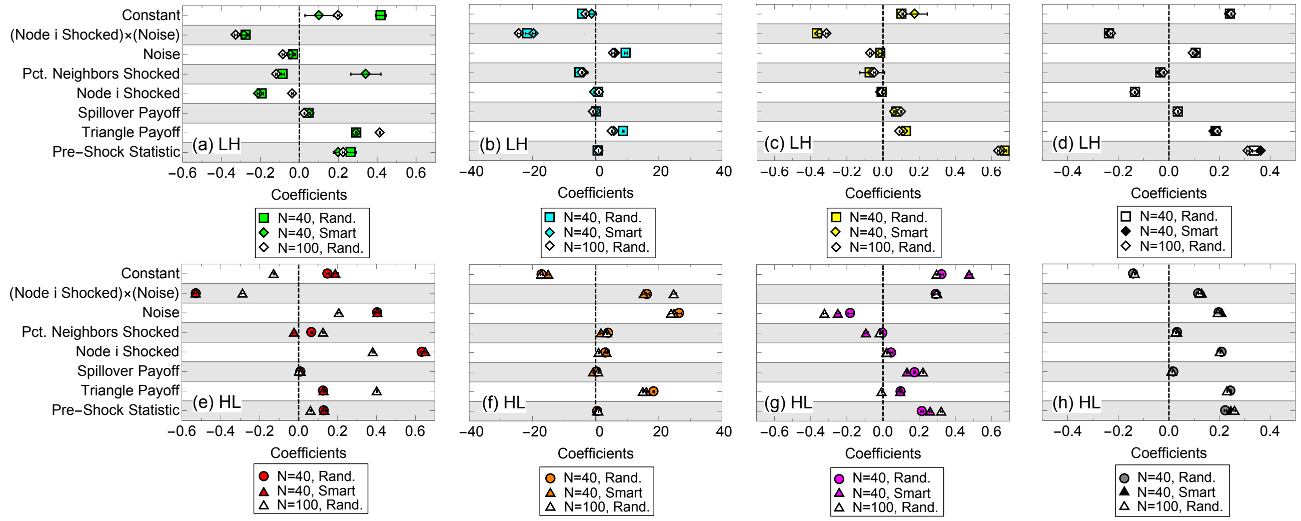

Figure S2. Comparisons of Determinants of Network Statistics given (1) Simulations with $N = 40$, random search, (2) Simulations with $N = 40$, "smart" search, and (3) Simulations with $N = 100$, random search. (a) LH clustering coefficient, (b) LH degree, (c) LH fraction spillover, (d) LH utility (normalized), (e) HL clustering coefficient, (f) HL degree, (g) HL fraction spillover, and (g) HL utility (normalized).

Condition	p	Mean	Std. Dev	N
LL	0	0	0	21,600
	0.25	66.39	254.73	21,600
	0.50	34.70	195.82	21,600
	0.75	11.93	96.63	21,600
HH	0	0	0	21,600
	0.25	8.08	29.98	21,600
	0.50	22.17	68.34	21,600
	0.75	40.62	131.95	21,600

Table S1. Differences between utility score at T100 (final time-point) and utility score at T50 (midway time-point) under different noise levels

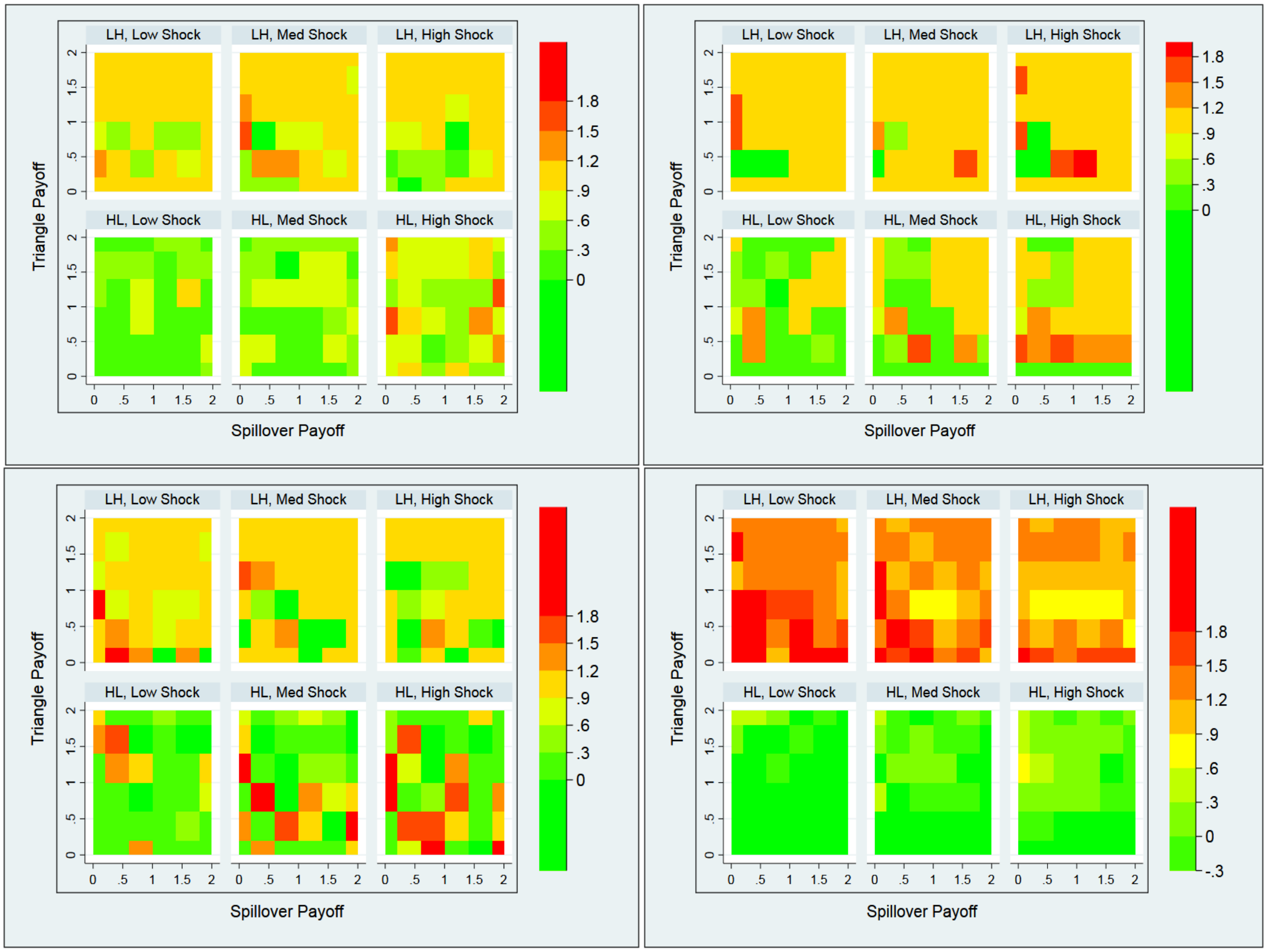

Figure S3. Network resilience and flexibility versus the number of nodes shocked ($p = 0$), where $N = 100$. Compare to main text Fig. 2. (a) degree resilience/flexibility, (b) clustering resilience/flexibility, (c) spillover resilience/flexibility, (d) utility resilience/flexibility. Low shock: 33 nodes shocked, medium shock: 66 nodes shocked, and high shock: all (100) nodes shocked.

References

1. Smaldino, P. E., D'Souza, R. & Maoz, Z. Resilience by structural entrenchment: Dynamics of single-layer and multiplex networks following sudden changes to the costs. *Netw. Sci.* (2017).

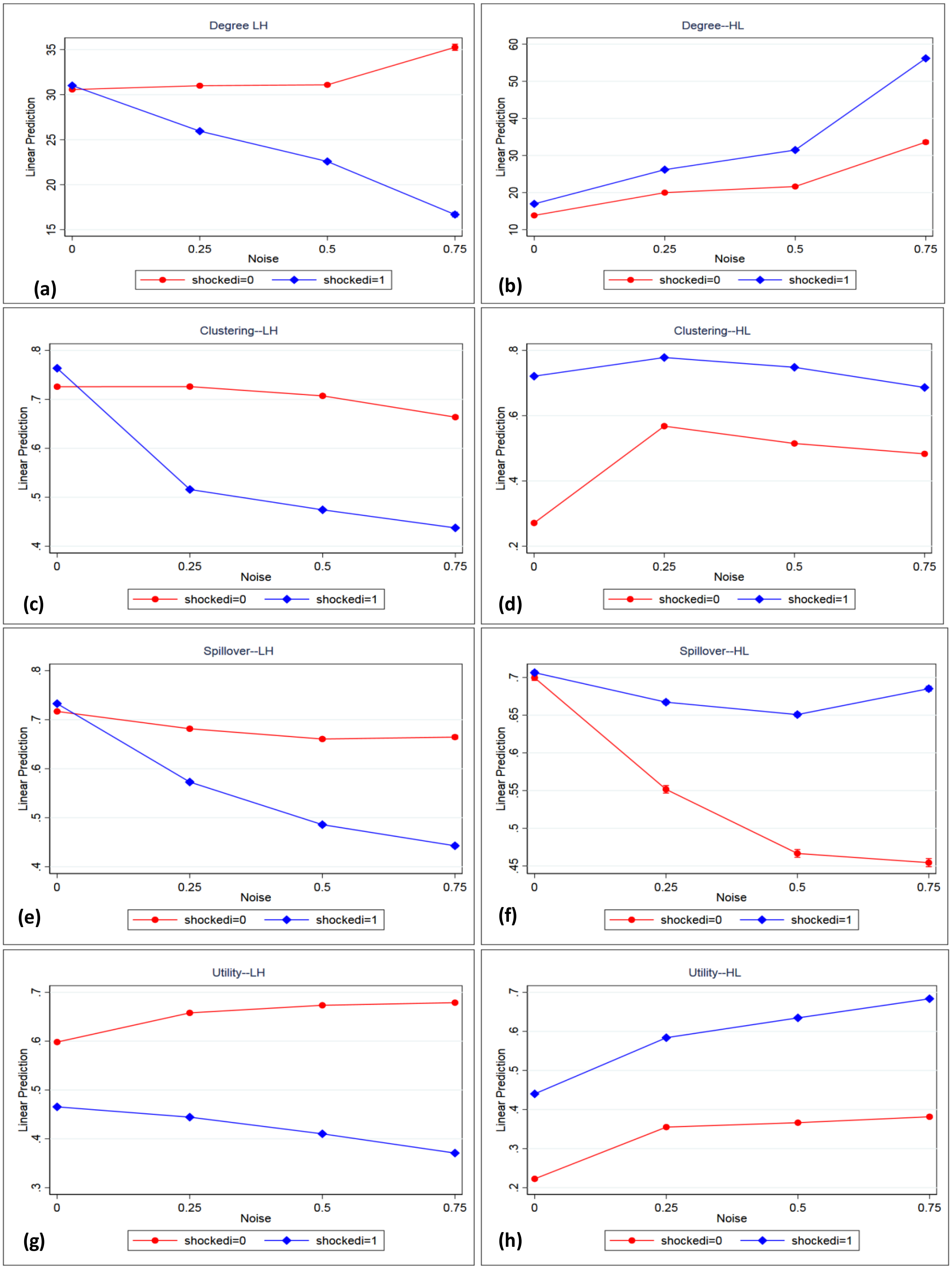

Figure S4. The effect of noise on degree (a–b), clustering (c–d), fraction spillover (e–f) and utility (g–h) for shocked and unshocked nodes, where 66 nodes are shocked (results are similar for other numbers of nodes shocked) and $N = 100$. Compare to main text Fig. 7. LH cost shocks are shown in the left panels, while HL cost shocks are shown in the right panels. Error bars are smaller than plot markers.
